# The physical interaction of p53 and plakoglobin is necessary for their synergistic inhibition of migration and invasion

**DOI:** 10.18632/oncotarget.8616

**Published:** 2016-04-06

**Authors:** Mahsa Alaee, Amarjot Padda, Vahedah Mehrabani, Lucas Churchill, Manijeh Pasdar

**Affiliations:** ^1^ Department of Oncology, University of Alberta, Edmonton, AB, T6G1Z2, Canada

**Keywords:** p53, plakoglobin, migration, invasion, tumor/metastasis suppressor

## Abstract

Plakoglobin (PG) is a paralog of β-catenin with similar adhesive, but contrasting signalling functions. Although β-catenin has well-known oncogenic function, PG generally acts as a tumor/metastasis suppressor by mechanisms that are just beginning to be deciphered. Previously, we showed that PG interacted with wild type (WT) and a number of mutant p53s, and that its tumor/metastasis suppressor activity may be mediated, at least partially, by this interaction. Here, carcinoma cell lines deficient in both p53 and PG (H1299), or expressing mutant p53 in the absence of PG (SCC9), were transfected with expression constructs encoding WT and different fragments and deletions of p53 and PG, individually or in pairs. Transfectants were characterized for their *in vitro* growth, migratory and invasive properties and for mapping the interacting domain of p53 and PG. We showed that when coexpressed, p53-WT and PG-WT cooperated to decrease growth, and acted synergistically to significantly reduce cell migration and invasion. The DNA-binding domain of p53 and C-terminal domain of PG mediated p53/PG interaction, and furthermore, the C-terminus of PG played a central role in the inhibition of invasion in association with p53.

## INTRODUCTION

The p53 transcription factor is a tumor suppressor that is absent or mutated in over half of all tumors [1-3]. p53 can be activated by various stress signals, including DNA damage, oncogenic insults, hypoxia, loss of cell-cell contact and changes in metabolic behavior. In response to stress, p53 activates physiological pathways that regulate cell cycle arrest, DNA repair, apoptosis, autophagy and metabolism [2, 3]. In addition to being a transcriptional regulator, p53 interacts with various cytoplasmic proteins, which mediate its growth regulating activity [4, 5].

The three structural domains [N-terminus (NT), DNA binding (DBD) and C-terminus (CT)] of p53 regulate its cellular functions. The NT contains two transactivation domains (TAD1 and 2). In addition to binding to coactivators, the NT is also the binding site for Hdm-2, which is an E3-ubiquitin ligase mediating p53 degradation, thus serving as the primary regulator of p53 levels [6, 7]. The CT contains an oligomerization domain, which allows p53 tetramerization, and a short regulatory domain, which may function as a non-specific DNA binding domain necessary for growth arrest and apoptosis [8, 9]. Flanked by the NT and CT, the DBD confers transcriptional activity on p53 and harbors the majority of p53 mutations [1, 10, 11]. p53 functions are regulated by posttranslational modifications and protein-protein interactions [5, 12, 13]. We have identified plakoglobin (PG, γ-catenin) as an endogenous interacting partner of both wild type (WT) and a number of mutant p53s, and have shown that PG's interaction with these mutants can restore their WT functions [14, 15].

PG is an Armadillo protein family member and a paralog of β-catenin with dual adhesive and signalling functions [16, 17, 18]. Structurally, these proteins consist of a N-terminal α-catenin binding domain, a core of Armadillo (Arm) repeats, which bind adhesive and signalling partners, and a TA domain [18]. In adherens junctions, both β-catenin and PG mediate cell-cell adhesion by interacting with classic cadherins and α-catenin, which link the complex to the cytoskeleton [18]. PG is also an essential desmosomal junction component and as such plays an integral role in cell-cell adhesion [18, 19]. Both β-catenin and PG affect cell signalling through interactions with intracellular partners involved in cell proliferation, differentiation, survival and apoptosis [18, 19]. Although β-catenin has a well-documented oncogenic function [18], PG is known to generally act as a tumor/metastasis suppressor by mechanisms that are beginning to be deciphered [19-22]. Our laboratory has shown that the tumor suppressor activity of PG, is, at least in part, mediated by its interaction with p53. We have shown that PG interacted with p53, and both were associated with the promoters of p53 target genes [e.g. *NME1*, *SFN* (14-3-3σ), *SATB1*, *THBS1*] [14, 15, 20]. Together, these results suggest that the tumor/metastasis suppressor activity of PG may be mediated by its interaction with p53 and regulation of p53 target genes.

In this study, we assessed the roles of p53 and PG, individually and together, in cell growth, migration and invasion, and identified the domains of p53 and PG that mediated their interaction. H1299 and SCC9 cells were cotransfected with expression constructs encoding HA-p53-(WT, NT, DBD and CT) and FLAG-PG-(WT, ΔN, ΔArm and ΔC). Transfectants were characterized for their growth, migration and invasion. p53/PG interaction and localization were determined by coimmunoprecipitation and confocal immunofluorescence microscopy. Our results suggested that 1) p53 and PG cooperated to decrease growth whereas they acted synergistically to significantly reduce migration and invasion of H1299 cells, 2) p53/PG interaction was mediated by the DBD of p53 and the C-terminus of PG, and 3) the C-terminal domain of PG was necessary for its maximum invasion inhibitory function via interaction with p53.

## RESULTS

### Reduced growth, migration and invasion of transfectants expressing p53, PG or p53 and PG

**Figure 1:**
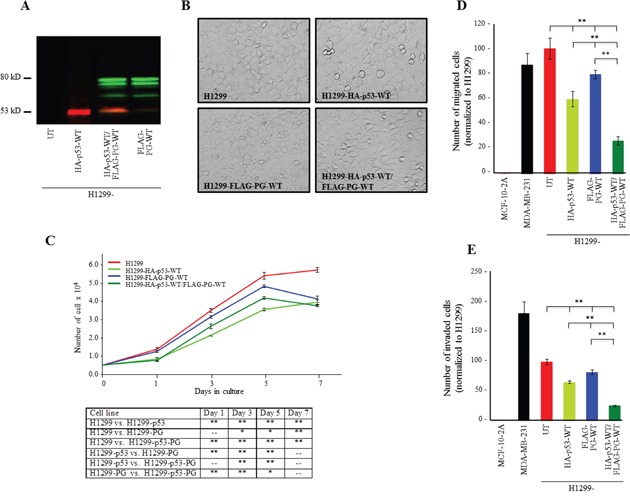
Growth, migration and invasion of H1299 cells expressing HA-p53-WT, FLAG-PG-WT or HA-p53-WT and FLAG-PG-WT **A.** Protein expression of HA-p53-WT and FLAG-PG-WT in H1299 cells. Total cell lysates from H1299 cells and H1299 cells transfected with HA-p53-WT, FLAG-PG-WT or both were processed for immunoblot with HA and FLAG antibodies at dilutions indicated in Table S2. **B.** Phase contrast micrograph (20x) of confluent cultures of H1299 and H1299-HA-p53-WT, FLAG-PG-WT or HA-p53-WT/FLAG-PG-WT. **C.** Untransfected (UT), HA-p53-WT or FLAG-PG-WT or HA-p53-WT/FLAG-PG-WT expressing H1299 cell were plated at single cell density (5×10^4^) in replicate cultures and allowed to grow for 7 days. At days 1, 3, 5, and 7 cultures were trypsinized and cells counted. Each time point represents the average of three independent experiments. The absence of error bars at some time points is due to the small differences among the experiments. **D.** Twenty-four-hour Transwell migration assays were performed in triplicate for the untransfected H1299 cells (UT) and H1299 transfectants expressing HA-p53-WT or FLAG-PG-WT or HA-p53-WT/FLAG-PG-WT. The membranes were fixed, stained, cut and mounted on slides and viewed under an inverted microscope. MCF-10-2A, a normal and MDA-MB-231, a highly invasive mammary epithelial cell lines were included in the assays as negative and positive controls, respectively. The number of migrated cells in five random fields for each membrane was calculated using the ImageJ Cell Counter program and averaged. Histograms represent the average ± SD of the number of migrated/invaded cells for each cell line. *p* values, * <0.05, ** < 0.001. **E.** Twenty-four-hour Matrigel invasion assays were performed as described in D using matrigel coated transwell membranes. PG, plakoglobin.

The expression of HA-p53-WT, FLAG-PG-WT and HA-p53-WT/FLAG-PG-WT in single and double transfectants was validated by western blot using anti-HA and anti-FLAG antibodies (Figure 1A) or p53 and PG antibodies (Figure S1). Figure 1B is a phase micrograph of confluent cultures of H1299 cells and its transfectants expressing HA-p53-WT, FLAG-PG-WT and HA-p53-WT/FLAG-PG-WT. Relative to H1299 cells, HA-p53-WT expressing transfectants were slightly larger and flatter. There were also some rounded, detached and presumably apoptotic cells in these cultures (H1299-HA-p53-WT). In contrast, FLAG-PG-WT cells appeared to form a tighter monolayer, consistent with the formation of adhesive junctions upon PG expression in these cells (H1299-FLAG-PG-WT). Interestingly, the double transfectants formed monolayers that were tighter than HA-p53-WT cells but not as tight as FLAG-PG-WT cells and furthermore showed some apoptotic cells (H1299-HA-p53-WT/FLAG-PG-WT) (Figure 1B).

The functional consequence of WT p53 and PG expression were assessed by examining the *in vitro* growth, migration and invasion of H1299 and H1299 transfectants (Figure 1C, 1D, 1E). Although the H1299-HA-p53 cells showed consistently and significantly less growth than H1299 cells (Figure 1C, H1299-HA-p53), the growth of H1299-FLAG-PG and H1299-HA-p53/FLAG-PG transfectants was similar to that of H1299 cells until day 5, when cultures became confluent and cell numbers sharply declined (Figure 1C, H1299-FLAG-PG, H1299-HA-p53/FLAG-PG). At day 7, H1299-HA-p53/FLAG-PG cells showed ~40% less growth than H1299 cells, whereas cells expressing either p53 or PG showed ~30% less growth (Figure 1C, Table 1).

**Table 1:** Summary of changes in the growth, migration and invasion of H1299 transfectants expressing various combinations of p53 and PG constructs

Cell line	% Decreased growth (day 7) Relative to H1299	% Decreased migration Relative to H1299	% Decreased invasion Relative to H1299
H1299-HA-p53-WT	32**	40**	34**
H1299-FLAG-PG-WT	28**	21**	18**
H1299-HA-p53-WT/FLAG-PG-WT	40**	73**	75**
H1299-FLAG-PG-WT/HA-p53-NT	9**	45**	12*
H1299-FLAG-PG-WT/HA-p53-DBD	10**	60**	12*
H1299-FLAG-PG-WT/HA-p53-CT	9**	45**	11*
H1299-HA-p53-WT/FLAG-PG-ΔN	35**	18**	67**
H1299-HA-p53-WT/FLAG-PG-ΔArm	31**	25**	70**
H1299-HA-p53-WT/FLAG-PG-ΔC	28**	29**	27**

*p* values, * <0.05, ** < 0.001.

Individual expression of either p53 or PG decreased migration by 40% and 21% relative to H1299 cells, respectively, whereas the coexpression of p53 and PG reduced migration by 73%. (Figure 1D, Table 1). Similarly, the invasiveness of H1299-HA-p53 and H1299-FLAG-PG cells was decreased by 35% and 21%, respectively, while the invasiveness of H1299-HA-p53/FLAG-PG cells was decreased by ~75% relative to H1299 cells (Figure 1D, Table 1). These results indicated that coexpression of p53 and PG synergistically and significantly decreased the migration and invasion of H1299 cells, and were also consistent with the reduced growth, migration and invasion of SCC9 cells upon the exogenous expression of PG [15, 23].

### Generation and characterization of cell lines expressing wild-type p53 and PG, various p53 fragments and PG deletion mutants

**Figure 2:**
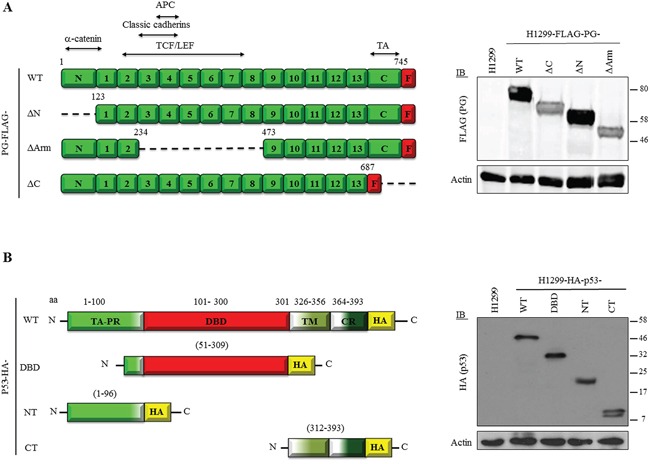
A. Expression of FLAG-tagged PG and HA-tagged p53 proteins in H1299 cells (Left) Domain structure of FLAG-tagged plakoglobin and plakoglobin deletion proteins. (Right) Expression of FLAG-tagged PG proteins in H1299 cells. H1299 cells were transfected with expression constructs encoding FLAG-tagged PG-WT, ΔN, ΔArm or ΔC. FLAG-PG-expressing stable cell lines were processed for immunoblot using FLAG and Actin (loading control) antibodies. APC, Adenomatous polyposis coli; TCF/LEF, T-cell factor/lymphoid enhancer factor; TA, Transactivation domain; F, FLAG tag. **B.** (Left) Domain structure HA-tagged p53 WT and deletion proteins. (Right) Expression of HA-tagged p53 proteins in H1299 cells. H1299 cells were transfected with constructs encoding HA-tagged p53-full length (WT), DNA binding domain (DBD), N-terminus (NT) and C-terminus (CT). p53-expressing stable cell lines were processed for immunoblot using HA and Actin (loading control) antibodies. TA, Transactivation; PR, proline-rich; TM, Tetramerization; CR, C-terminal regulatory domain; HA, HA tag.

To identify the domains of p53 and PG mediating their interactions, we created constructs encoding various deletions of FLAG-tagged PG, and constructs encoding different fragments of HA-tagged p53 (Figure 2). The PG constructs have been described previously [23, 24] and include PG-WT (a.a. 1-745), -ΔN (a.a. 123-745; lacking the α-catenin binding domain), -ΔArm [a.a. 1-216 and 464-745; lacking Armadillo domains 3-7, involved in binding to classic cadherins and adenomatous polyposis coli)] and -ΔC (a.a. 687-745; lacking the TA domain). All PG constructs contained a C-terminal FLAG tag (Figure 2A, left), and were previously characterized in SCC9 cells [24]. These constructs were transfected into H1299 cells and their expression was verified by immunoblotting with FLAG antibodies (Figure 2A, right).

Constructs encoding C-terminally HA-tagged WT and fragments of p53 were generated, including p53-WT (a.a. 1-393), -NT [a.a. 1-96; containing both TAs (a.a. 1-42; 43-92), the nuclear export signal (a.a. 11-27) and the proline-rich domain (a.a. 64-92)], -DBD [a.a. 51-309; including the second TAD, proline-rich domain, and entire DBD (a.a. 101-300)], and -CT [a.a. 312-393; containing the 3 nuclear localization sequences (a.a. 305-322; 369-375; 379-384), tetramerization domain (a.a. 326-356), and regulatory domain (a.a. 364-393)] (Figure 2B, left). The HA-p53 constructs were transfected into H1299 cells and protein expression was confirmed by immunoblotting with HA antibodies (Figure 2B, right).

### Expression of HA-p53 and FLAG-PG proteins in H1299 double transfectants

**Figure 3:**
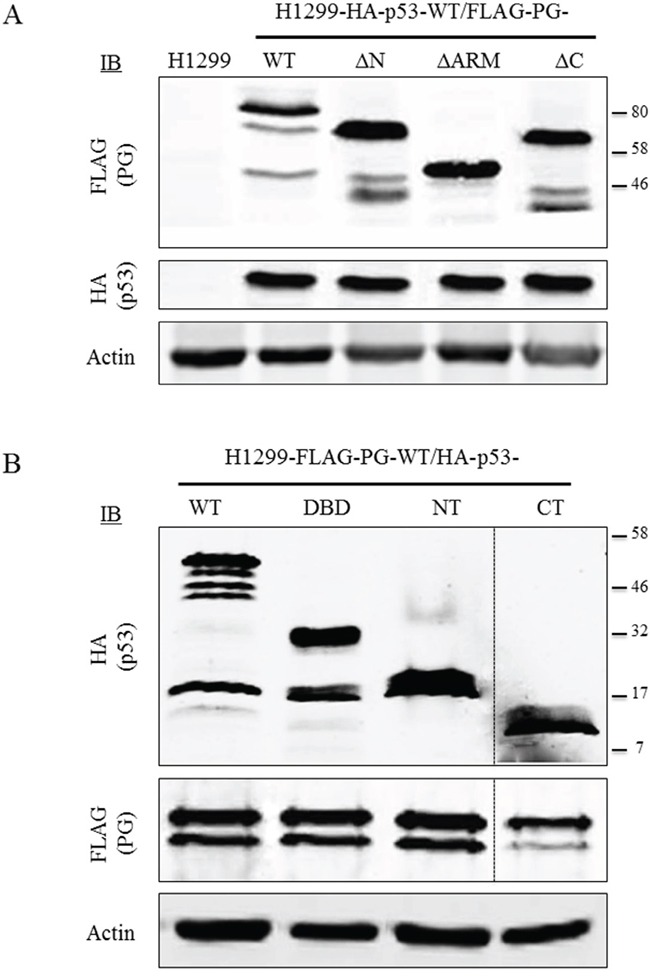
Protein expression of WT and fragments of p53 and PG in double transfectants Equal amounts of total cellular proteins from stable H1299-HA-p53-WT transfectants coexpressing FLAG-PG-WT, - ΔN, -ΔArm or -ΔC **(A)** or H1299-FLAG-PG-WT coexpressing HA-p53 WT, -NT, -DBD or -CT **(B)** were processed for immunoblots with HA or FLAG antibodies as described in Materials and Methods. PG, plakoglobin; WT, wild type; N, N-terminus; C, C-terminus; Arm, armadillo; DBD, DNA binding domain.

To study p53 and PG interaction, we generated H1299 or SCC9 double transfectants coexpressing HA-p53-WT with FLAG-PG-WT, -ΔN, -ΔArm or -ΔC or -FLAG-PG-WT with HA-p53-WT, -NT, -DBD or -CT. Protein expression in H1299 (Figure 3A, 3B) and SCC9 (Figure S2) double transfectants was confirmed by immunoblotting with HA and FLAG antibodies (Figure 3 and Figure S2).

### DNA binding domain of p53 and the C-terminal domain of PG mediate p53/PG interactions

**Figure 4:**
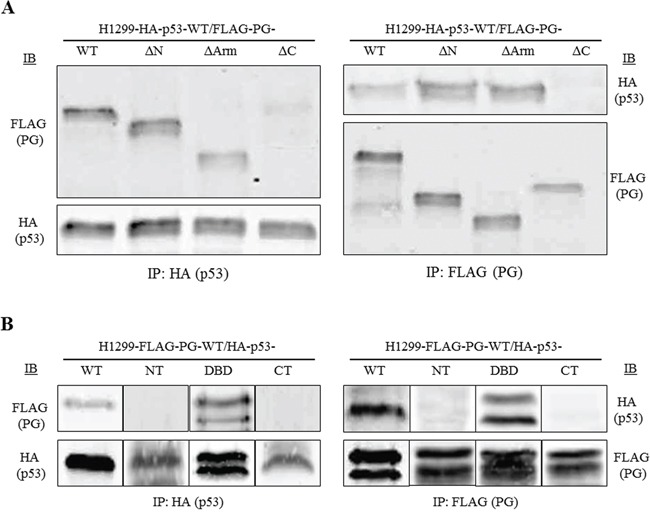
DNA binding domain of p53 interacts with the C-terminal domain of PG Equal amounts of total cell extracts from double transfectants coexpressing HA-p53-WT and various FLAG-tagged PG proteins **(A)** or FLAG-PG-WT and various HA-tagged p53 proteins **(B)** were processed for reciprocal and sequential immunoprecipitation and immunoblotting using HA and FLAG antibodies as described in Materials and Methods. The immune complexes in A were separated on 7.5%, and in B on 5-20% SDS gradient gels. PG, plakoglobin; WT, wild type; N, N-terminus; C, C-terminus; Arm, Armadillo; DBD, DNA binding domain.

H1299 double transfectants coexpressing various pairs of HA-p53 and FLAG-PG proteins/fragments were processed for reciprocal coimmunoprecipitation and immunoblotting with HA and FLAG antibodies. Figure 4A shows the coimmunoprecipitation results with H1299 cells expressing HA-p53-WT together with FLAG-PG-WT, -ΔN, -ΔArm or -ΔC. In lysates from these transfectants, FLAG antibodies coprecipitated HA-p53-WT with FLAG-PG-WT, -ΔN and -ΔArm, but not with FLAG-PG-ΔC. The reciprocal coimmunoprecipitation using HA antibodies confirmed these findings, as FLAG-PG-ΔC was the only FLAG-PG fragment that was not coprecipitated with HA-p53-WT. These results suggested that the C-terminus domain of PG is necessary for p53/PG interactions (Figure 4A). When H1299 cells expressing FLAG-PG-WT with HA-p53-WT, -NT, -DBD or -CT were subjected to reciprocal coimmunoprecipitation, FLAG antibodies coprecipitated HA-p53-WT and -DBD, but not HA-p53-NT or -CT (Figure 4B). These results were confirmed when HA antibodies coprecipitated FLAG-PG-WT with HA-p53-DBD, but not HA-p53-NT or -CT (Figure 4B). Taken together, these results suggest that the C-terminus of PG, and the DBD of p53 mediate p53/PG interaction.

### Subcellular location of p53 and PG in H1299-HA-p53 and H1299-FLAG-PG transfectants

**Figure 5:**
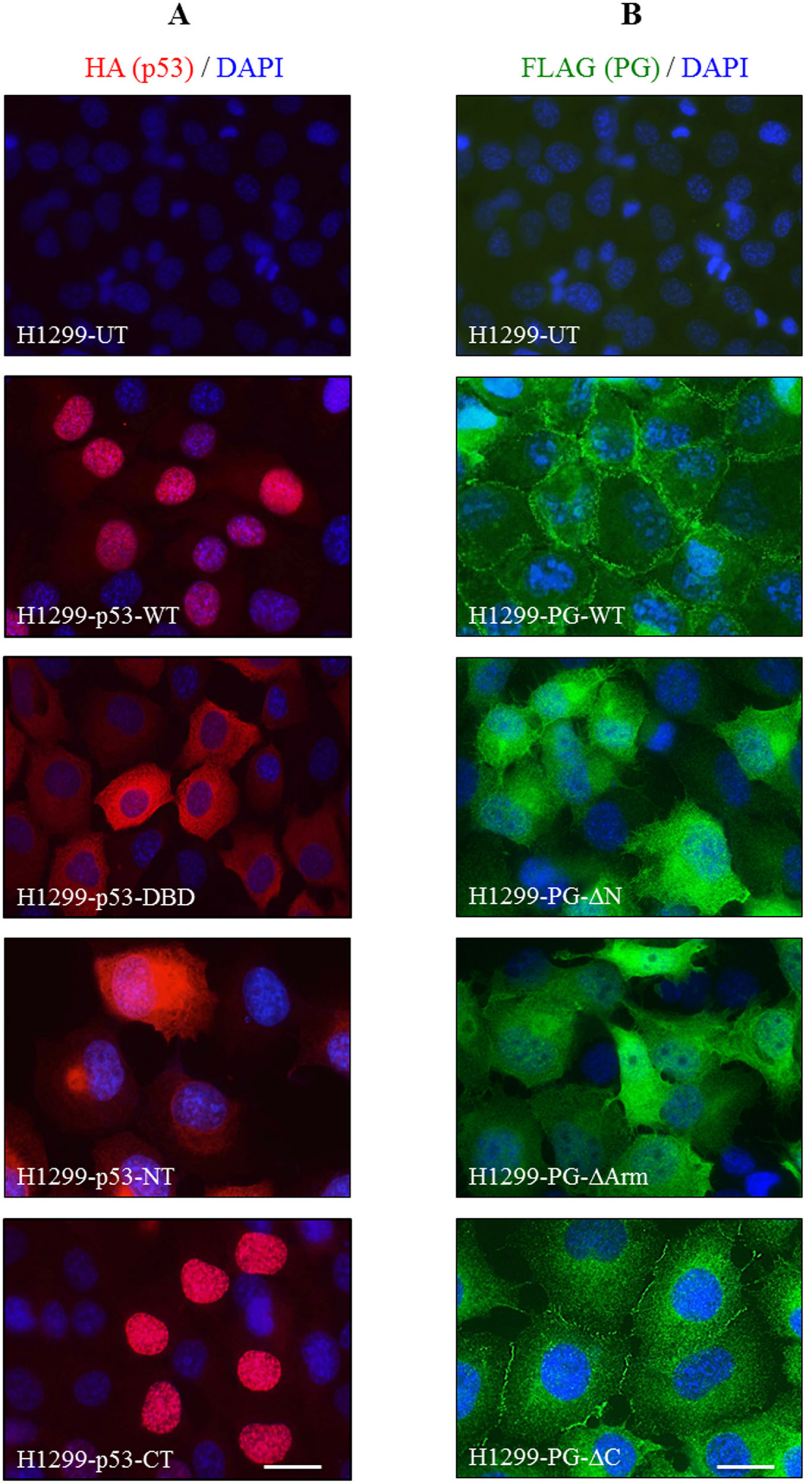
Subcellular localization of HA-tagged p53 (A) and FLAG-tagged PG (B) proteins in H1299 cells H1299 cells expressing various FLAG-PG and HA-p53 proteins were grown to confluency on coverslips, fixed with formaldehyde and permeabilized with CSK buffer. Coverslips were processed for confocal immunofluorescence microscopy using FLAG (green) and HA (red) antibodies. Nuclei were counterstained with DAPI (blue) and coverslips mounted and viewed using a Zeiss confocal microscope. PG, plakoglobin; WT, wild type; N, N-terminus; C, C-terminus; Arm, Armadillo; DBD, DNA binding domain. Bar, 40 μm.

We previously demonstrated that p53 and PG interacted in both the cytoplasm and nucleus [14]. Here, HA-p53 and FLAG-PG transfectants were processed for immunofluorescence using HA and FLAG antibodies. Figure 5A shows the subcellular localization of p53 in various H1299-HA-p53 transfectants. In HA-p53-WT transfectants, p53 was primarily nuclear, with a faint cytoplasmic distribution (Figure 5A, H1299-HA-p53-WT). In contrast, p53 was distributed mainly in the cytoplasm of H1299-HA-p53-DBD transfectants with very little nuclear staining (Figure 5A, H1299-HA-p53-DBD). In H1299-HA-p53-NT transfectants, p53 was mainly cytoplasmic, with a distinct peri-nuclear distribution (Figure 5A, H1299-HA-p53-NT). Finally, in HA-p53-CT transfectants, p53 was detected exclusively in the nucleus, resembling the HA-p53-WT transfectants (Figure 5A, H1299-HA-p53-CT). Collectively, these results are consistent with the presence of the nuclear localization sequence in p53-WT and -CT, and its absence in p53-DBD and -NT.

H1299 cells expressing FLAG-PG-WT or its three deletions showed different PG staining and cell morphology (Figure 5B). H1299-FLAG-PG-WT transfectants exhibited typical epithelial morphology and extensive cell-cell contact, with PG localized primarily to the areas of cell-cell contact (Figure 5B, H1299-FLAG-PG-WT). H1299-FLAG-PG-ΔN and H1299-FLAG-PG-ΔArm transfectants had numerous processes and little cell-cell contact, consistent with these fragments lacking the ability to interact with α-catenin and cadherins and localize to adhesive junctions. In these transfectants, PG-ΔN and PG-ΔArm were mainly detected throughout the cytoplasm, without any distinct membrane staining (Figure 5B, H1299-FLAG-PG-ΔN, -FLAG-PG-ΔArm). In contrast, FLAG-PG-ΔC transfectants showed epithelial morphology, but were flatter than H1299-FLAG-PG-WT cells. In these cells, PG-ΔC was localized to the areas of cell-cell contact and cytoplasm, but was clearly excluded from the nucleus (Figure 5B, H1299-FLAG-PG-ΔC). Together, these results suggest that the C-terminus of PG may be necessary for its nuclear localization.

### Subcellular distribution of PG and p53 in H1299 double transfectants expressing FLAG-PG-WT and HA-p53-WT, -NT, -DBD or -CT

**Figure 6:**
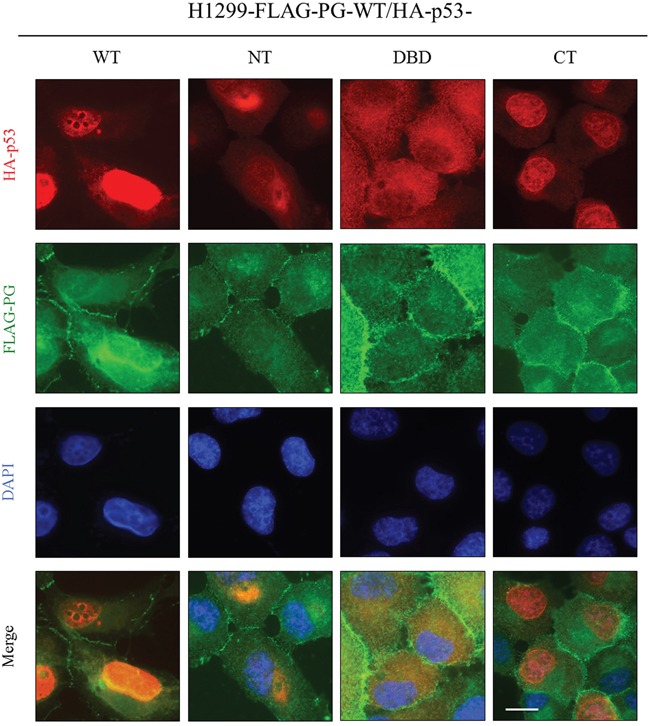
Subcellular localization of PG and p53 in H1299 double transfectants coexpressing FLAG-PG-WT and HA-p53-WT, -NT, -DBD or -CT Cultures were processed for double immunofluorescence with FLAG and HA antibodies as described in the legend of Figure 5. WT, wild type; PG, plakoglobin; NT, N-terminus; CT, C-terminus; DBD, DNA binding domain. Bar, 25 μm.

In HA-p53-WT and FLAG-PG-WT cotransfectants, p53 was primarily nuclear with faint cytoplasmic staining, whereas PG was localized to the areas of cell-cell contact as well as in the cytoplasm and nucleus. There was an overlap between the nuclear p53 and the nuclear PG staining in these cells (Figure 6, H1299-FLAG-PG-WT/HA-p53-WT). Membrane and cytoplasmic distribution of PG was also detected in H1299-FLAG-PG-WT/HA-p53-NT transfectants, in which p53-NT distribution was almost exclusively cytoplasmic/perinuclear, overlapping with the cytoplasmic PG staining. Nuclear PG was not detected in these cells (Figure 6, H1299-FLAG-PG-WT/HA-p53-NT). In H1299-FLAG-PG-WT/HA-p53-DBD cells, PG was primarily membrane localized, whereas p53-DBD was primarily cytoplasmic and overlapped with a pool of cytoplasmic PG (Figure 6, H1299-FLAG-PG-WT/HA-p53-DBD). FLAG-PG-WT/HA-p53-CT transfectants showed membrane localization of PG with some homogeneous cytoplasmic staining, whereas p53-CT was almost exclusively nuclear. No overlap was detectable in the distribution of the two proteins (Figure 6, H1299-FLAG-PG-WT/HA-p53-CT). These observations are consistent with the presence of nuclear localization signals in p53-CT and suggest that PG was codistributed only with the p53-WT and with p53-DBD (albeit in the cytoplasm).

### Subcellular distribution of PG and p53 in SCC9 double transfectants expressing HA-p53-WT and FLAG-PG-WT, -ΔN, -ΔArm or -ΔC

**Figure 7:**
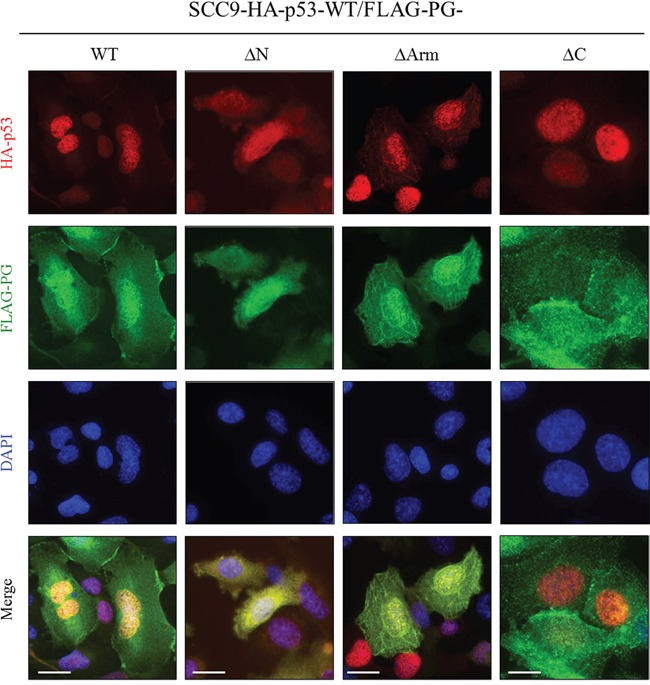
Subcellular localization of PG and p53 in SCC9 double transfectants coexpressing HA-p53-WT and FLAG-PG-WT, -ΔN, -ΔArm or -ΔC **A.** Cultures were processed for double immunofluorescence with FLAG and HA antibodies as described in the legend of Figure 5. WT, wild type; PG, plakoglobin; N, N-terminus; C, C-terminus; Arm, Armadillo. Bar, 25 μm (HA-p53-WT and FLAG-PG-WT, -ΔN, -ΔArm) and 15 μm (HA-p53-WT and FLAG-PG -ΔC).

In SCC9 cells expressing HA-p53-WT and FLAG-PG-WT, the distribution of p53 and PG was similar to that of H1299-FLAG-PG-WT/HA-p53-WT cells. PG was detected at the membrane, and in the cytoplasm and nucleus. Nuclear PG was codistributed with p53, which was almost exclusively nuclear (Figure 7, SCC9-HA-p53-WT/FLAG-PG-WT). In the HA-p53-WT/FLAG-PG-ΔN transfectants, PG-ΔN was detected throughout the cells, overlapping in distribution with p53, which was detected in both the cytoplasm and nucleus (Figure 7, SCC9-HA-p53-WT/FLAG-PG-ΔN). In HA-p53-WT/FLAG-PG-ΔArm transfectants, PG-ΔArm was detected throughout the cell, while p53 was primarily nuclear with some cytoplasmic distribution. In these cells, p53 was codistributed with PG-ΔArm in both the cytoplasm and nucleus (Figure 7, SCC9-HA-p53-WT/FLAG-PG-ΔArm). In contrast to the FLAG-PG-WT, -ΔN or -ΔArm transfectants in which PG was detected in the nucleus, FLAG-PG-ΔC transfectants had no detectable nuclear PG-ΔC. Due to the exclusively nuclear distribution of p53 in these cells, no overlap of p53 and PG-ΔC was detected (Figure 7, SCC9-HA-p53-WT/FLAG-PG-ΔC). Collectively, these results suggested that the C-terminus of PG is necessary for its localization to the nucleus and its colocalization with p53.

### Cooperation of p53 and PG in regulating growth, migration and invasion of H1299 cells

We also investigated the role of various structural domains of p53 and PG in their combined inhibition of the growth, migration and invasion of H1299 cells. *In vitro* growth assays showed a small reduction (~10%) in the growth of transfectants expressing FLAG-PG-WT and p53-NT, -DBD or -CT compared to H1299 cells. In comparison, the growth of H1299-HA-p53-WT/FLAG-PG-WT cells was reduced by ~40% (Figure S3; Table 1). In contrast, the growth of H1299 cells expressing HA-p53-WT and FLAG-PG-ΔN, -ΔArm or -ΔC was the same or slightly less than H1299-HA-p53-WT/FLAG-PG-WT cells (Figure S3; Table 1).

**Figure 8:**
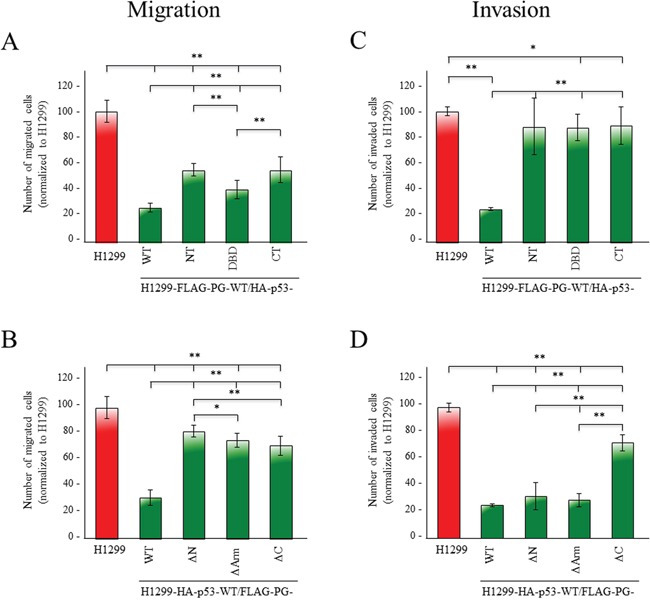
Contribution of various p53 and PG domains to their synergistic inhibition of migration and invasion H1299 and H1299 transfectants expressing FLAG-PG-WT and HA-p53-WT, -NT, -DBD, or -CT **(A, C)** or HA-p53-WT and FLAG-PG-WT, -ΔN, -ΔArm or -ΔC **B, D.** were processed for migration (A, B) and invasion (C, D) assays as described in the legends to Figure 1C. All values were normalized to H1299 cells. *p* values, * <0.05, ** <0.001.

Figure 8A shows the effect of various p53 domains on cell migration in a FLAG-PG-WT background. The coexpression of HA-p53-WT and FLAG-PG-WT reduced the migration of H1299 cells by >70% compared to parental H1299 cells (Figures 1B, 8A, Table 1). Cells coexpressing FLAG-PG-WT and various HA-p53 fragments (H1299-FLAG-PG-WT/p53-NT, -DBD, -CT) were more migratory than H1299-FLAG-PG-WT/p53-WT cells, but significantly less than H1299 cells (Figure 8A, Table 1). Among the fragments, HA-p53-DBD transfectants were less migratory than HA-p53-NT or CT transfectants, which had similar migration levels (Figure 8A, Table 1).

In a HA-p53-WT background, while cells expressing FLAG-PG deletions were less migratory than H1299 cells, they were significantly more migratory than FLAG-PG-WT transfectants. When compared, H1299-HA-p53-WT/−FLAG-PG-WT double transfectants were >70% less migratory than H1299 cells. H1299-HA-p53-WT/−FLAG-PG-ΔN, -ΔArm, -ΔC double transfectants showed reduced migration by 18%, 25% and 29%, respectively (Figure 1B, 8B, Table 1).

Invasion assays showed that H1299-HA-p53-WT/FLAG-PG-WT double transfectants, were 75% less invasive than H1299 cells (Figure 1C, 8C, Table 1). The expression of any of the HA-p53 fragments in a FLAG-PG-WT background (H1299-FLAG-PG-WT/p53-NT, -DBD, -CT) showed increased invasiveness (Figure 8C, Table 1), although these transfectants were still less invasive than the H1299 cells (Figure 8C, Table 1).

Finally, HA-p53-WT/FLAG-PG-ΔN and -ΔArm double transfectants showed a decrease in invasiveness that was comparable to the FLAG-PG-WT transfectants (67% and 70% vs. 73%), whereas HA-p53-WT/FLAG-PG-ΔC transfectants were significantly more invasive (27% vs. 74%) (Figure 8D, Table 1).

Together, the results in Figures 1, 8 and S2, and Table 1 suggested that: 1) individual expression of either p53 or PG reduced the growth, migration and invasion of H1299, 2) p53 alone was more effective than PG alone, 3) the greatest reduction was attained when both proteins were expressed, 4) the PG C-terminus domain was necessary for the inhibition of invasion.

## DISCUSSION

We showed that p53 and PG cooperatively reduced growth and acted synergistically to decrease cellular migration and invasion. The two proteins interacted with each other via the DNA-binding domain of p53 and the transactivation domain of PG.

p53 prevents cancer development and progression by transcriptionally regulating genes involved in cell cycle arrest, senescence and cell death/apoptosis [25, 26]. p53 also has transcription/nuclear-independent growth inhibitory functions, the most well-characterized of which is the induction of apoptosis [4, 27-31].

H1299-HA-p53-WT transfectants showed significantly lower growth, migration and invasion. These effects are mediated by the p53 regulation of expression of various tumor suppressors, signaling molecules and oncogenic and tumor suppressor miRNAs [32-40]. p53 also promotes stable junction formation and cadherin-mediated contact inhibition by downregulating transcriptional repressors of E-cadherin and regulating cytoskeleton remodeling [41-44].

H1299-FLAG-PG-WT cells also showed significant reductions in growth, migration and invasion. Unlike H1299-HA-p53 transfectants in which growth was decreased from day 1, the H1299-FLAG-PG cells showed similar growth kinetics to that of H1299 cells until day 5, when cell numbers declined due to the induction of contact inhibition of growth. PG can also suppress tumor growth by inducing apoptosis [46-48]. These findings are consistent with the role of PG as an essential regulator of cell-cell adhesion and growth [19, 45-48].

Numerous *in vitro* and *in vivo* studies have shown that PG has tumor/metastasis suppressor activities. The loss of heterozygosity and low frequency mutations in the PG gene was shown to predispose patients to familial breast and ovarian cancers [49]. PG knockdown in human umbilical vein endothelial cells promoted migration, tubular formation and angiogenesis [50]. Since these early studies, PG's role in the inhibition of migration and invasion has been demonstrated in many carcinoma cell lines [15, 19, 51-55]. Paralleling these *in vitro* observations, loss/changes in PG levels and localization are associated with increased metastasis and poor prognosis *in vivo* [19].

PG also acts as a tumor/metastasis suppressor independent of its role in cell-cell adhesion. PG null keratinocytes expressing exogenous PG-WT, -ΔN or -ΔC showed similar adhesiveness but different migratory properties. Although PG-WT and -ΔN transfectants were not migratory, PG-ΔC transfectants became migratory via activation of Src signaling [53], suggesting that the TA is essential for the tumor/metastasis suppressor activity of PG. PG may regulate gene expression independent of its role in cell-cell adhesion via interaction with transcription factors including TCF/LEF, CBP, SOX4 and p53 [14, 21, 56-61]. We previously showed that PG interacted with both WT and several mutant p53s in various carcinoma cell lines, leading to the induction of a non-transformed phenotype. This phenotypic transition coincided with changes in the expression of several p53 target genes, the promoters of which interacted with both p53 and PG [14, 15]. Recently, Sechler *et al.* (2015) reported that PG overexpression in NSCLC cells reduced cell migration via HAI-1 induction, in a p53-dependent manner [22]. These observations are consistent with the dramatic decreases in the migration and invasion of H1299-HA-p53-WT/FLAG-PG-WT cotransfectants vs. cells expressing either HA-p53 or FLAG-PG alone [15, 23, 55].

Coimmunoprecipitation experiments revealed that p53 interacted with the TA domain of PG via its DBD. Immunofluorescence staining showed colocalization of FLAG-PG-WT and HA-p53-DBD within the cytoplasm, consistent with the absence of nuclear localization signal in p53-DBD. Similar experiments with cells expressing p53-WT and various PG deletions showed a lack of interaction between p53 and PG-ΔC. In HA-p53-WT-FLAG-PG-ΔC cells, PG distribution was primarily at the membrane, whereas p53 was exclusively nuclear, further confirming that PG interacted with p53 via its C-terminal domain.

We also examined the changes in growth, migration and invasion of H1299 cells coexpressing various HA-p53 fragments with FLAG-PG-WT or various FLAG-PG deletions with HA-p53-WT. These results showed that only cells coexpressing p53-WT and PG-WT exhibited maximum inhibition of cell growth, migration and invasion. This finding is novel and has not been previously reported. In contrast, the coexpression of HA-p53-NT, -DBD and -CT with FLAG-PG-WT reduced cell growth and invasiveness by only ~10-12%. Interestingly, however, all p53 fragments were effective in reducing the migration of H1299 double transfectants noticeably, albeit not to the level of p53-WT.

The NT domain regulates the p53-mediated transcription via interaction with the basal transcription machinery, but also has transcription-independent functions. The NT also regulates the stability of p53 by binding to Hdm-2, and its regulation of growth by interactions with apoptotic proteins and FAK [29, 62-65]. However, both the DBD and the CT are necessary for proper functioning of the NT domain [66-74], consistent with the limited capacity of NT to reduce the growth and invasiveness of H1299 transfectants observed in our study.

The DBD construct used in this study also includes the TAD2 domain. The DBD has a tightly regulated, sequence-specific DNA binding activity and plays a critical role in p53 transcriptional activity and also mediates the cytosolic function of p53 in regulating apoptosis [5, 71, 75]. Here, we showed that DBD plus TAD2, which is involved in senescence induction [64], is not sufficient to significantly reduce the growth and invasiveness of H1299 transfectants.

H1299-HA-p53-CT cells expressed a peptide comprising the oligomerization and transcriptional regulatory domains [70, 76-79]. The CT domain contains many phosphorylation and acetylation sites which confer the proper conformation, localization, stability, DNA binding and transcriptional activity on p53 [5, 74, 80-83]. Our data showed almost exclusive nuclear localization of p53-CT, while p53-NT and -DBD proteins were localized entirely within the cytoplasm. However, while properly localized, the CT domain alone was not sufficient to reduce the growth and invasiveness of H1299 cells to the same extent as WT-p53.

Surprisingly, the coexpression of p53-NT, -DBD or -CT with PG-WT decreased the migratory properties of the respective H1299 transfectants, albeit to a lesser extent than p53-WT. A number of studies have shown interactions between NT, DBD and CT with various kinases involved in migration including FAK, JNK, PLK1 and GSK3β [38, 51, 84-90]. Our results clearly suggest that the NT, DBD and CT fragments of p53 retain some ability to inhibit cell migration. Whether the expressed fragments could act as dominant negative peptides to sequester these kinases is not clear and warrants further investigation.

In a p53-WT background, various PG deletions exhibited reduced growth similar to H1299-HA-p53 cells, suggesting that the inhibition of growth by PG was primarily mediated by its role in the induction of contact inhibition. Moreover, p53 may have a larger contribution to the significantly reduced growth of H1299 cells coexpressing p53 and PG.

When PG deletions were coexpressed with p53-WT, these transfectants were less migratory than H1299 cells (~25% reduction). However, their migration was significantly higher than H1299-HA-p53-WT/FLAG-PG-WT cells (~75% reduction). This is consistent with the inability of PG-ΔN and ΔArm to interact with α-catenin and cadherins, respectively, mediate stable junction formation and inhibit migration. However, while PG- ΔC expressing cells exhibited extensive cell-cell contact, they also showed increased migration. This observation is also in keeping with previous studies demonstrating the involvement of the C-terminal domain of PG in inhibition of migration independent of its adhesive properties [53]. Consistent with this observation, PG- ΔC expressing cells exhibited extensive cell-cell contact, but increased migration. The invasiveness of H1299-HA-p53-WT/FLAG-PG- ΔN and - ΔArm (with intact TA domain) was similar to that of H1299-HA-p53-WT/FLAG-PG-WT cells (~70%), whereas invasiveness was reduced by only ~27% in H1299-HA-p53-WT/FLAG-PG- ΔC. These results may be explained by the loss of interaction between PG and p53 due to the absence of TA domain of PG.

In conclusion, our data indicated that 1) p53 and PG cooperated to reduce the growth and acted synergistically to decrease migration and invasiveness of H1299 cells and 2) the C-terminal domain of PG interacted with the DBD of p53, and this interaction was necessary for the maximum inhibition of invasion by p53 and PG. The data presented also raises the possibilities that the NT, CT and DBD fragments of p53 may act in a dominant negative manner to inhibit signaling pathways involved in migration. Furthermore, the differences in the migratory properties of the transfectants expressing various p53 fragments relative to the WTp53 cells may suggest that the genes/pathways involved in inhibition of migration by p53 may be different than those involved in its inhibition of growth and invasion. Future studies will be focused on determining the exact amino acids involved in p53/PG interactions and examining the interactions between p53 fragments and various signaling molecules that regulate cell migration. Since more than 50% of all tumors and 80% of metastatic tumors have mutations in p53 [1], our observations provide the exciting possibility that PG may be a potential therapeutic target for cancers with non-functional mutant p53s.

## MATERIALS AND METHODS

### Reagents, cells and culture conditions

Chemical reagents were purchased from Sigma-Aldrich (Oakville, Canada) and tissue culture reagents from Invitrogen (Burlington, Canada), unless stated otherwise. Dr. Roger Leng, University of Alberta, provided the p53 and PG null non-small cell lung carcinoma cell line H1299 [91]. The p53 mutant and PG deficient human tongue squamous cell carcinoma cell line SCC9 has been described [23, 24]. All cells were maintained in Minimum Essential Medium (MEM) supplemented with 10% fetal bovine serum (FBS), and 1% penicillin-streptomycin-kanamycin (PSK) antibiotics.

### Plasmid construction and transfection

The FLAG-tagged PG (-WT, -ΔN, -ΔArm, -ΔC) constructs and their SCC9 transfectants have been described [24]. A plasmid encoding WT-p53 (PGEX2TK-WT-p53, gift from Dr. Roger Leng) served as the template for constructing HA-tagged p53 WT, and p53 fragments, NT, DBD, and CT.

Various primers (Table S1) were used to generate the four p53 inserts by PCR. The PCR products were then subcloned into pcDNA 3.1 containing an HA tag at the C-terminus. The pcDNA 3.1 vector was modified with the HA epitope tag sequence (TAC CCA TAC GAT GTT CCA GAT TAC GCT), which contained restriction sites to facilitate the subcloning of the p53 inserts and a stop codon. The constructs encoding HA-tagged p53-WT, NT, DBD, or CT (Figure 2B) were verified by sequencing.

H1299 or SCC9 cells cultured in 60 mm dishes or on glass coverslips were transfected at 60-80% confluency with 2-10 μg of DNA. Twenty hours later, cells were rinsed and allowed to recover for 24 hour in complete MEM. For transient transfections, transfected cells were processed for different assays 48 hour after transfection. For stable transfectants, 48 hour after transfection, media were replaced with media containing 500 μg/ml hygromycin B (p53) or 400 μg/ml G418 (PG) and the resistant colonies selected for 2-3 weeks and verified for HA-p53 and FLAG-PG expression. Positive clones were subcultured by limiting dilution and maintained in media containing 350 μg/ml hygromycin B and 200 μg/ml G418.

### Preparation of total cell extracts and immunoblotting

Confluent 100 mm culture dishes were rinsed with cold PBS, solubilized in hot SDS sample buffer (10 mM Tris-HCl pH 6.8, 2% (w/v) SDS, 50 mM dithiothreitol (DTT), 2 mM EDTA, 0.5 mM PMSF) and boiled for 10 minutes. Twenty-five - 50 μg of total cellular protein were resolved by SDS-PAGE, transferred to nitrocellulose membranes and processed for immunoblotting using HA, FLAG and actin primary antibodies followed by the appropriate secondary antibodies (Table S2). Membranes were developed by either ECL (Perkin Elmer LAS) or LI-COR IR fluorescence dyes.

### Immunoprecipitation

Confluent cultures in 100 mm plates were rinsed with cold PBS containing 1mM NaF, Na_3_VO_4_ and CaCl_2_ and extracted in 2 ml of lysis buffer (50 mM Tris-HCl pH 7.5, 150mM NaCl, 1% NP-40, 0.5% sodium deoxycholate, 0.7 μg/ml Pepstatin, 1 mM Na_3_VO_4_, 1 mM NaF, and protease inhibitor cocktail) for 30 minutes at 4°C on a rocker. Cells were scraped and centrifuged at 48000xg for 10 minutes. Supernatants were divided into equal aliquots and processed for immunoprecipitation with FLAG and HA antibodies (Table S2) and 40 μ l protein G agarose (for monoclonal antibodies) or protein A sepharose beads (Pierce Biotechnology, IL, USA) for polyclonal antibodies) beads (Pierce Biotechnology, IL, USA) overnight at 4°C on a rocker-rotator. Samples were then centrifuges at 14000xg for 2 minutes to separate the beads from the supernatants and the supernatants were processed for a second immunoprecipation for 2-3 hours. Beads from the two immunoprecipitations were combined and washed three times with the lysis buffer. Immune complexes were solubilized in 40 μl SDS sample buffer, separated by PAGE and processed for immunoblot using HA, FLAG and actin primary antibodies followed by the appropriate secondary antibodies (Table S2) as described above.

### Immunofluorescence

Cells were grown to confluency on glass coverslips and rinsed twice with cold PBS containing 1mM NaF, Na_3_VO_4_ and CaCl_2_. Cells were then fixed with 3.7% formaldehyde for 20 minutes and extracted with CSK buffer (50mM NaCl, 300 mM Sucrose, 10 mM PIPES pH 6.8, 3 mM MgCl_2_, 0.5% Triton X-100, 1.2 mM PMSF, and 1 mg/ml DNase and RNase;) for 7 minutes. Coverslips were blocked with 4.0% goat serum and 50mM NH_4_Cl_4_ in PBS containing 0.2% BSA (PBS–BSA) for 1 hour and processed for indirect immunofluorescence. Coverslips were incubated in the primary antibodies followed by the species-specific secondary antibodies at concentrations indicated in Table S2 for 1 hour and 20 minutes, respectively. All antibodies were diluted in PBS–BSA. Nuclei were counterstained with DAPI (1:2,000) in PBS. Coverslips were mounted in elvanol containing 0.2% (w/v) paraphenylene diamine (PPD) and viewed using a Zeiss confocal microscope.

### *In vitro* growth, migration and invasion assays

For growth assays, triplicate cultures of various cell lines were plated in 24-well plates at single cell density (2.5 × 10^4^/cm^2^). At 1, 3, 5 and 7 days after plating, cultures were trypsinized and cells counted. Each time point represents the average of three independent experiments.

For cell migration assays, 2×10^5^ cells were resuspended in 500 μ l serum-free media and plated in the upper chamber of transwell inserts (3μm pore, 6.5mm diameter; BD Biosciences, CA, USA). Normal media containing 10% FBS was added to the lower chamber. Cultures were incubated at 37°C in 5% CO_2_ for 24 hour to allow cell migration. Inserts were transferred into new dishes and rinsed with PBS to remove un-attached cells. Inserts were fixed with 3.7% formaldehyde (in PBS) for 2 minutes, permeabilized with 100% methanol for 20 minutes and stained with Giemsa stain for 15 minutes at room temperature. Following staining, membranes were cut, mounted using permount (Fisher, Canada), viewed under an inverted microscope using a 20x objective lens and photographed. The migrated cells on the underside of the membranes were counted in 5 random fields from the photographs.

Matrigel invasion assays were performed according to the manufacturer's protocol (BD Biosciences). Cells were starved in serum free media 24 hour prior to plating. For each cell line, 5×10^4^ cells in 0.2ml serum-free media were plated in the top compartment of Matrigel-coated invasion chambers (8 μm pore PETE membrane). Fibroblast conditioned media (0.8ml) was added to the bottom chambers and plates were incubated overnight at 37°C in 5% CO_2_. After 24 hour, membranes were recovered and processed as described for the migration assay. Mounted membranes were viewed under a 20x objective lens of an inverted microscope and photographed. The invaded cells were counted in 5 random fields for each membrane.

ImageJ Cell Counter program was used to calculate the numbers of migrated/invaded cells. Counted cell numbers were averaged and histograms were constructed after normalizing the average numbers of migrated/invaded cells in each transfected cell line to those of their parental untransfected cells. Each assay was repeated 2-5 independent times.

### Statistical analysis

Values are presented as means±SD. Statistical differences between groups were assessed by Student's t-tests. All experiments were performed at least three times. P-values <0.05 were considered significant.

## SUPPLEMENTARY FIGURES AND TABLES


