# Supplementary material for: The physical interaction of p53 and plakoglobin is necessary for their synergistic inhibition of migration and invasion
Source: Oncotarget. 2016 Apr 6;7(18):26898–915. doi: 10.18632/oncotarget.8616 (PMC5042024; doi:10.18632/oncotarget.8616)
Supplement: Supplementary file 1 [file oncotarget-07-26898-s001.pdf]

SUPPLEMENTARY FIGURES AND TABLES

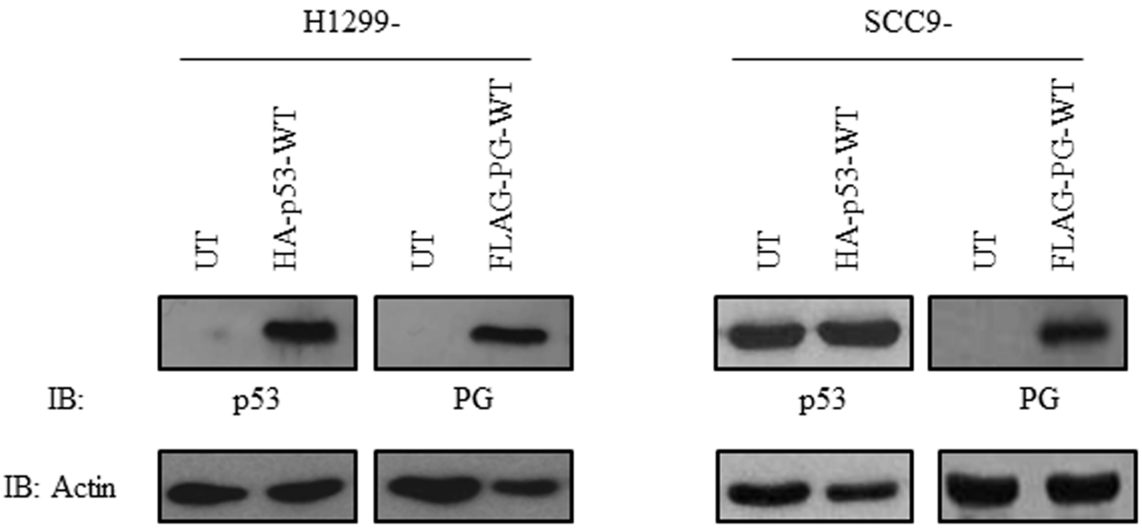

Supplementary Figure S1: Protein expression of HA-p53-WT and FLAG-PG-WT in H1299 cells. Total cell lysates from untransfected (UT) H1299 and SCC9 cells and their transfectants expressing HA-p53-WT or FLAG-PG-WT were processed for immunoblotting with anti-p53, anti-plakoglobin or anti-actin antibodies. PG, plakoglobin.

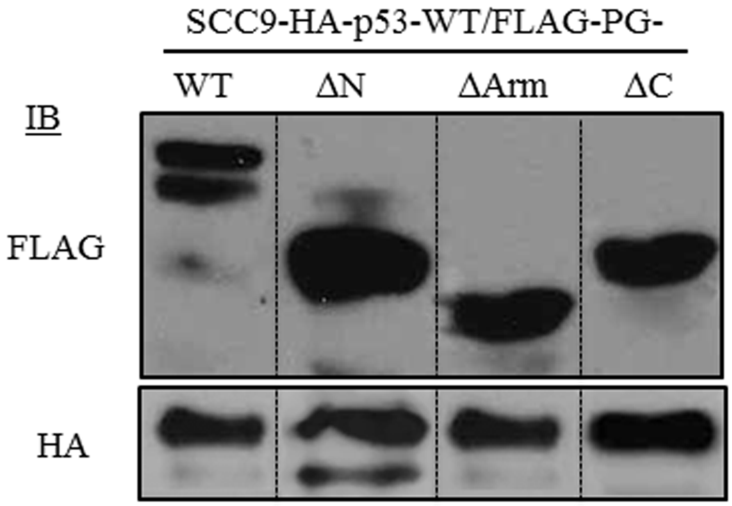

Supplementary Figure S2: Protein expression of HA-p53-WT and FLAG-PG-WT, -DN , -DArm and -DC in SCC9 cells. Total cell lysates from SCC9 cells expressing HA-p53-WT and FLAG-PG-WT, -ΔN, ΔArm or -ΔC were processed for immunoblots with FLAG and HA antibodies.

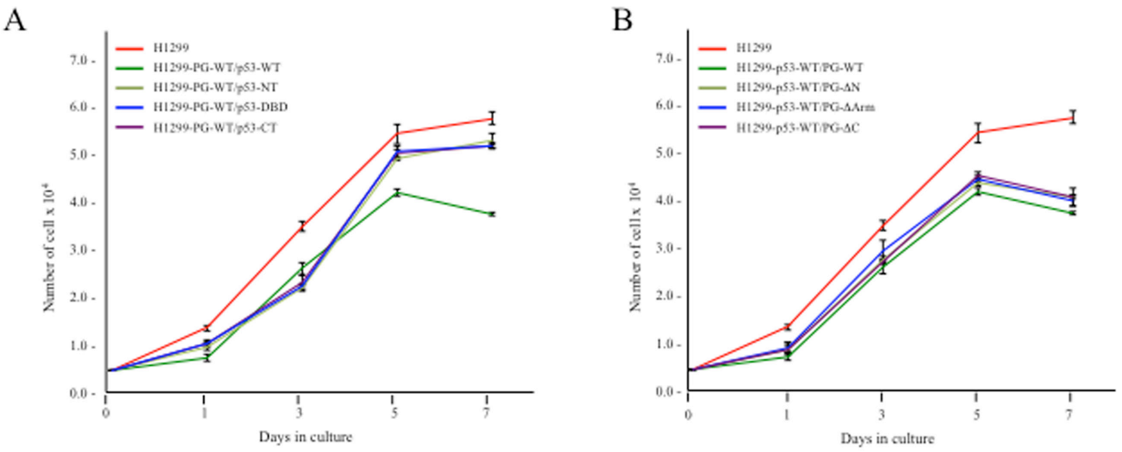

Cell line	Day			
	1	3	5	7
H1299 vs. H1299-p53-WT/PG-WT	**	*	*	**
H1299 vs. H1299-PG-WT/p53-NT	*	**	*	*
H1299 vs. H1299-PG-WT/p53-DBD	*	*	*	*
H1299 vs. H1299-PG-WT/p53-CT	*	**	*	*

Cell line	Day			
	1	3	5	7
H1299 vs. H1299-p53-WT/PG-WT	**	*	*	**
H1299 vs. H1299-p53-WT/PG-ΔN	*	**	*	*
H1299 vs. H1299- p53-WT/PG-ΔArm	*	*	*	**
H1299 vs. H1299- p53-WT/PG-ΔC	*	*	*	**

Supplementary Figure S3: Contribution of various p53 and PG domains to their growth inhibitory function. H1299 and H1299 transfectants coexpressing PG-WT and p53-WT, NT, -DBD, or -CT **(A)** or p53-WT and PG-WT, -ΔN, -ΔArm or -ΔC **(B)** were plated at single cell density (5 X 104) in replicate cultures and allowed to grow for 7 days. At days 1, 3, 5, and 7 cultures were trypsinized and cells counted. Each time point represents the average of three independent experiments. The absence of error bars at some time points is due to the small differences among the experiments. * <0.05, ** < 0.001.

Supplementary Table S1: Oligos/primers sequences used to create p53 constructs

Construct		Primers	Size (bp)
p53-WT	Forward	5’ ttt taa gct tat gga gga gcc gca gtc ag 3’	29
	Reverse	5’ ttt tgc ggc cgc gtc tga gtc agg ccc ttc tgt c 3’	34
P53-NT	Forward	5’ ttt taa gct tat gga gga gcc gca gtc ag 3’	29
	Reverse	5’ ttt tgc ggc cgc agg agc tgc tgg tgc agg 3’	30
P53-DBD	Forward	5’ ttt taa gct tat gtc cca agc aat gga tga ttt g 3’	34
	Reverse	5’ ttt tgc ggc cgc ccc ttt ctt gcg gag att ctc 3’	33
P53-CT	Forward	5’ ttt taa gct tat gac cag ctc ctc tcc cca gc 3’	32
	Reverse	5’ ttt tgc ggc cgc gtc tga gtc agg ccc ttc tgt c 3’	34
HA tag	Forward	5’ ttt gct agc atg gcg gcc gca tac cca tac gat gtt cca gat 3’	42
	Reverse	5’ aaa tct aga cta aag ctt agc gta atc tgg aac atc gta 3’	39

For all primers, pre-denaturation was done at 95°C for 2 minutes followed by 32 cycles of denaturation at 95°C for 30 seconds, annealing at 50°C (first 7 cycles) and 55°C (last 25 cycles) for 30 seconds, and extension at 72°C for 90 seconds.

Supplementary Table S2: Antibodies and their respective dilutions in specific assays

Primary antibodies	Species	Assay			Company/Catalog number
		WB	IP	IF	
Anti-HA	Rat	1:500	1:150	1:100	Roche/11867423001
Anti-β-Actin	Mouse	1:2000	--	--	Santa Cruz/sc-47778
Anti-FLAG	Mouse	1:500	1:150	1:100	Sigma/F-3165
**Secondary antibodies**					
HRP anti-mouse, IgG light chain	Goat	1:2000	--	--	Jackson/115-005-174
HRP anti-rat, IgG light chain	Goat	1:2000	--	--	Jackson/112-005-175
Alexa Fluor anti-mouse IgG, light chain	Goat	1:25000	--	--	Jackson/112-625-175
Alexa Fluor anti-rat IgG, light chain	Goat	1:25000	--	--	Jackson/115-625-174
Alexa 488 anti-mouse IgG	Goat	--	--	1:1000	Molecular probes/A11029
Rhodamine/TRITC anti-Rat IgG	Rabbit	--	--	1:1000	Sigma/T4280
